# Correction to “Toward an Effective Translational Science Engine”

**DOI:** 10.1111/cts.70219

**Published:** 2025-04-02

Ioachimescu OC, Shaker R. Toward an Effective Translational Science Engine. *Clin Transl Sci*. 2024 Nov;17(11):e70069. doi: 10.1111/cts.70069. PMID: 39539022; PMCID: PMC11561134.

The funding statement should read “NIH (NCATS) UL1 TR001436 (MCW IRB# FP00015891)” rather than “CTSA: FP00015891.”

The corresponding author's email address should be changed to oioachimescu@mcw.edu.

Affiliation 1 should be Milwaukee, Wisconsin, not Hartland, Wisconsin.

The address for the corresponding author should be 8701 Watertown Plank Rd., Suite M1350, Milwaukee, WI 53226, USA.

We apologize for this error.

